# Supplementary material for: Computational Assessment of Blood Flow Heterogeneity in Peritoneal Dialysis Patients' Cardiac Ventricles
Source: Front Physiol. 2018 May 17;9:511. doi: 10.3389/fphys.2018.00511 (PMC5968396; doi:10.3389/fphys.2018.00511)
Supplement: Supplementary file 1 [file Data_Sheet_1.pdf]

## Supplementary Methods and Results

# Computational assessment of blood flow heterogeneity in peritoneal dialysis patients' cardiac ventricles

Sanjay R Kharche [1,3*], Aaron So [2, 3], Fabio Salerno [1], Ting-Yim Lee [2], Chris Ellis [3], Daniel Goldman [3], C W McIntyre [1,3*]

[1] Kidney Clinical Research Unit, Victoria Hospital, Lawson's Health Research Institute, London, N6A 5W9, Ontario, Canada.
[2] Robarts Research Institute, University of Western Ontario, London, N6A 5B7, Ontario, Canada.
[3] Department of Medical Biophysics, Schulich School of Medicine and Dentistry, University of Western Ontario, London, Ontario, N6A 5C1, Canada.

Corresponding authors:

Drs Sanjay R Kharche and Christopher W McIntyre
Room ELL 112, Kidney Clinical Research Unit
800 Commissioners Road, Victoria Hospital
Lawson's Health Research Institute
Medical Biophysics, University of Western Ontario
London Ontario N6A 5W9, Canada
Email: Sanjay.Kharche@lhsc.on.ca
C.W.McIntyre@lhsc.on.ca

# A. SUPPLEMENTARY METHODS

## Section S1. Imaging protocol.

*S1.1 CT imaging:* At each scan, an intravenous injection of Iopamidol (Isovue 370) contrast agent at 5 mL/s at a dosage of 0.7 mgI/kg followed by saline flush at the same injection rate. Dynamic contrast-enhanced (DCE) scanning of the heart were performed at rest and during maximal coronary vasodilation stress induced by adenosine administration at 140 µg/kg per minute for 3 minutes. A total of four scans were acquired for each patient during two visits (2 scans at each visit). During the first visit, rest / stress DCE acquisition was performed when the patient was given a warmed (37ºC) dialysate. The scans were repeated during the second visit when the patient was given a cooled (32ºC) dialysate.

*S1.2 CT image acquisition:* Prior to scanning, the patient was laid on the scanner table in a supine position and heart rate reduced using a beta blocker. Dynamic contrast-enhanced (DCE) CT images of the heart were acquired at 20 to 25 mid-to-end diastoles (heart rate dependent) with breath-hold using a GE Healthcare Revolution CT scanner immediately following a bolus injection of iodinated contrast agent as described in section 2.1.2. The acquisition parameters were set to 100 kV tube voltage, 100 mA tube current, 280 ms gantry period and 80 mm axial coverage. DCE heart images were reconstructed at 5 mm slice thickness and registered to each other using a three-dimensional non-rigid registration algorithm. Parametric perfusion maps were then generated from the registered DCE images with a proprietary software (CT Perfusion, GE Healthcare) run on a workstation. The raw imaging data set consisted of 16 images with an in-plane resolution of approximately 0.5 mm x 0.5 mm, and inter-plane resolution of 5 mm. For the three patients, a total of 12 data sets were obtained. The DCE heart images were anonymized and transferred to a workstation for generating perfusion maps.

*S1.3 Method for computing myocardial blood flow maps from DCE CT images:* DCE heart images were analysed with the CT Perfusion software (GE Healthcare) on a workstation. The arterial and myocardial time-density curves obtained from the dynamic contrast enhanced CT images of the heart were analysed with a model-based deconvolution, from which myocardial blood flow (BF, perfusion) was derived in units of ml/min/mg.

## Section S2. Bi-ventricular human ventricle geometry as truncated ellipsoids.

*S2.1 Myocardial tissue between ellipsoidal surfaces:* The left ventricle (LV) shape can be approximated with truncated ellipsoids (Mercier et al., 1982). The right ventricle (RV) can also be represented as a truncated ellipsoid. The generic heart myocardial tissue geometry is defined by the radii of ellipsoids encompassing epicardial and endocardial surfaces, and the height of the truncating basal plane, which in our case was taken to be $z = 0$. The equation of an ellipsoid in Cartesian coordinates is

$$\frac{(x-x_c)^2}{a^2}+\frac{(y-y_c)^2}{b^2}+\frac{(z-z_c)^2}{c^2}=1 \quad \text{Equation S1}$$

where $O=(x_c, y_c, z_c)$ is an arbitrarily chosen origin by the user. For the LV epicardial surface $a=b=30\ mm$, and $c=70\ mm$ (see Figure S1); for the LV endocardial surface $a=b=18\ mm$, and $c=58\ mm$. LV myocardium is defined as the volume between these two surfaces. In case of the RV epicardium, we take $a=24\ mm$, $b=45\ mm$ and $c=54\ mm$. Both the LV and the RV are truncated with the geometry defined only for $0 \le z$. In addition, whenever the RV ellipsoids overlapped with the LV or its chamber, they were ignored since that is already defined as LV.

*S2.2 Normal to ellipsoidal surface:* An ellipsoidal surface

$$S=\frac{(x-x_c)^2}{a^2}+\frac{(y-y_c)^2}{b^2}+\frac{(z-z_c)^2}{c^2}-1 \quad \text{Equation S2}$$

has normal along the following vector

$$\vec{n}=\vec{\nabla}S=\frac{2(x-x_c)}{a^2}\hat{i}+\frac{2(y-y_c)}{b^2}\hat{j}+\frac{2(z-z_c)}{c^2}\hat{k} \quad \text{Equation S3}$$

$\vec{n}$ is taken going away from the origin, *O*, on the epicardial surface and towards on the endocardial surface. The septum is treated as having two endocardial surfaces and no epicardial surface. The normal was divided by its magnitude to obtain the unit normal for each surface.

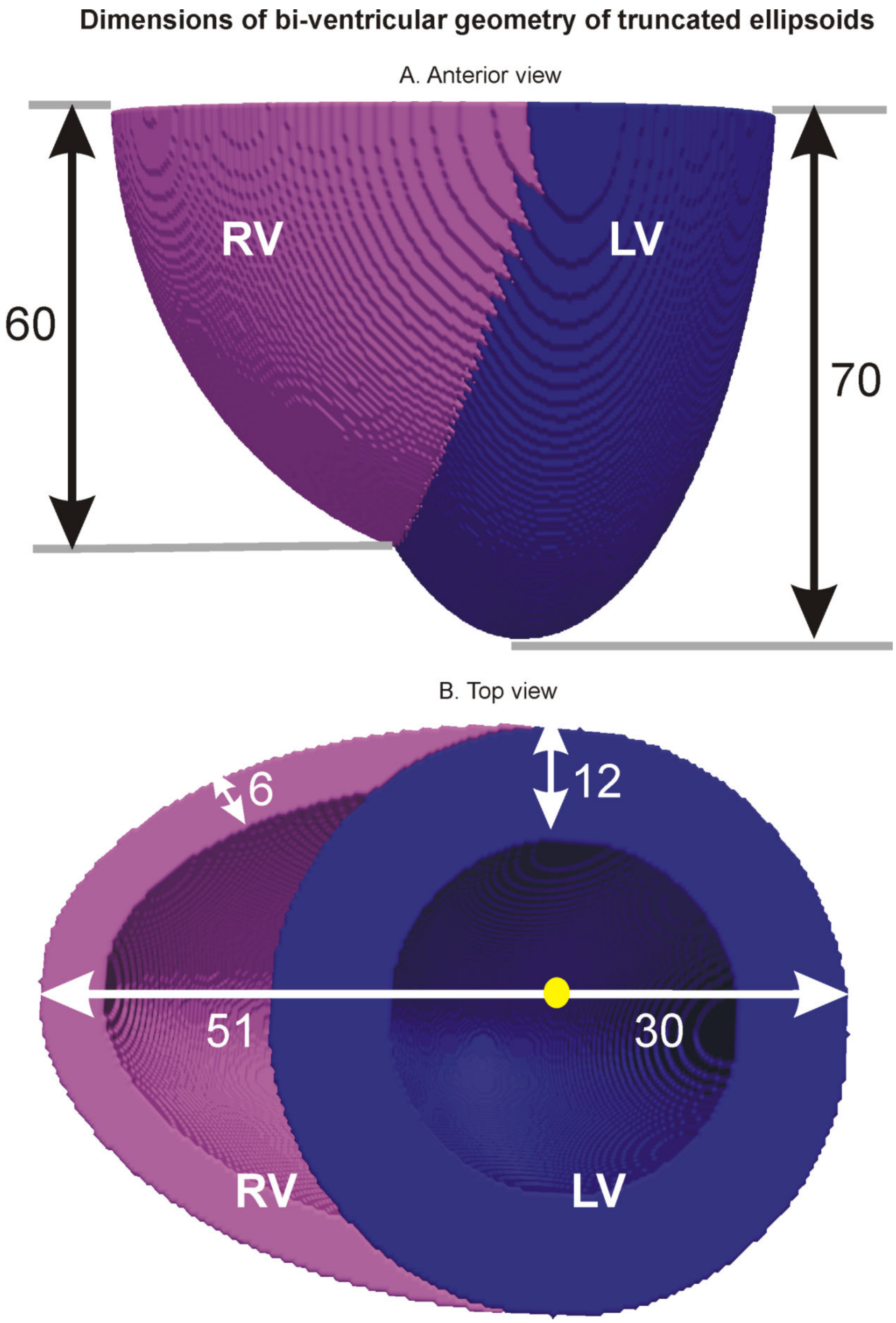


**Figure. S1.** Anatomy dimensions. The template heart geometry constructed using truncated ellipsoids. (see Supplementary Section S2 for details).

**Table S3. Kassab *et al.* (Kassab et al., 1993) morphometry data for RCA, LAD, and LCX used in the model.**

S3a. Diameters and lengths of coronary vessels in RCA, LAD, and LCX.

| | **Elements** | | | | | |
|---|---|---|---|---|---|---|
| **Order** | **RCA** | | **LAD** | | **LCX** | |
| | **Diameter (μm)** | **Length (mm)** | **Diameter (μm)** | **Length (mm)** | **Diameter (μm)** | **Length (mm)** |
| **1** | 9.3 | 0.125 ± 0.084 | 9.0 | 0.115 ± 0.066 | 9.0 | 0.115 ± 0.066 |
| **2** | 12.8 | 0.141 ± 0.103 | 12.3 | 0.136 ± 0.088 | 12.3 | 0.136 ± 0.088 |
| **3** | 17.7 | 0.178 ± 0.105 | 17.7 | 0.149 ± 0.104 | 17.7 | 0.149 ± 0.104 |
| **4** | 28.6 | 0.253 ± 0.174 | 30.5 | 0.353 ± 0.154 | 27.5 | 0.405 ± 0.170 |
| **5** | 63.1 | 0.545 ± 0.415 | 66.2 | 0.502 ± 0.349 | 73.2 | 0.908 ± 0.763 |
| **6** | 132 | 1.64 ± 1.13 | 139 | 1.31 ± 0.914 | 139 | 1.83 ± 1.34 |
| **7** | 256 | 3.13 ± 2.11 | 308 | 3.54 ± 2.11 | 279 | 4.22 ± 2.26 |
| **8** | 428 | 5.99 ± 3.53 | 462 | 4.99 ± 3.02 | 462 | 6.98 ± 3.92 |
| **9** | 706 | 9.06 ± 5.56 | 714 | 9.03 ± 6.13 | 961 | 21.0 ± 15.6 |
| **10** | 1302 | 16.1 ± 13.3 | 1573 | 20.3 ± 17.9 | 2603 | 49.6 |
| **11** | 3218 | 84.1 | 3176 | 47.9 | | |

S3b. Segment to element ratios.

| Order | Segment-to-element ratio | | |
|---|---|---|---|
| | RCA | LAD | LCX |
| 1 | 1.88 ± 0.99 | 2.30 ± 1.4 | 2.30 ± 1.4 |
| 2 | 1.88 ± 1.0 | 1.79 ± 0.95 | 1.79 ± 0.95 |
| 3 | 2.20 ± 1.2 | 2.00 ± 1.1 | 2.00 ± 1.1 |
| 4 | 2.30 ± 1.8 | 2.28 ± 1.3 | 2.06 ± 1.2 |
| 5 | 2.00 ± 0.91 | 2.02 ± 1.2 | 2.20 ± 1.3 |
| 6 | 2.30 ± 1.3 | 2.23 ± 1.3 | 2.11 ± 1.1 |
| 7 | 3.23 ± 2.1 | 3.89 ± 2.1 | 2.75 ± 1.6 |
| 8 | 4.68 ± 2.7 | 4.69 ± 3.0 | 4.22 ± 2.4 |
| 9 | 5.38 ± 3.6 | 6.06 ± 4.2 | 6.60 ± 4.0 |
| 10 | 8.50 ± 7.2 | 9.0 ± 7.0 | 14 |
| 11 | 26 | 17 | n/a |

S3c. Connectivity matrices for RCA, LAD, and LCX coronary arterial trees.

RCA

| Order n | Order m (parent segment) | | | | | | | | | | | |
|---|---|---|---|---|---|---|---|---|---|---|---|---|
| | 1 | 2 | 3 | 4 | 5 | 6 | 7 | 8 | 9 | 10 | 11 | 12 |
| 0 | 0 | 0.955 | 0.234 | 0.048 | 0.012 | 0 | 0 | 0 | 0 | 0 | 0 | 0 |
| 1 | 0 | 0.045 | 0.739 | 0.253 | 0.090 | 0.002 | 0.001 | 0 | 0 | 0 | 0 | 0 |
| 2 | 0 | 0 | 0.028 | 0.678 | 0.211 | 0.048 | 0.006 | 0.008 | 0 | 0 | 0 | 0 |
| 3 | 0 | 0 | 0 | 0.022 | 0.639 | 0.207 | 0.093 | 0.053 | 0.024 | 0.009 | 0 | 0 |

| | | | | | | | | | | | | |
|---|---|---|---|---|---|---|---|---|---|---|---|---|
| 4 | 0 | 0 | 0 | 0 | 0.048 | 0.640 | 0.131 | 0.139 | 0.079 | 0.047 | 0 | 0 |
| 5 | 0 | 0 | 0 | 0 | 0 | 0.103 | 0.719 | 0.309 | 0.255 | 0.125 | 0.072 | 0 |
| 6 | 0 | 0 | 0 | 0 | 0 | 0 | 0.049 | 0.463 | 0.232 | 0.246 | 0.178 | 0.032 |
| 7 | 0 | 0 | 0 | 0 | 0 | 0 | 0 | 0.028 | 0.393 | 0.180 | 0.276 | 0.355 |
| 8 | 0 | 0 | 0 | 0 | 0 | 0 | 0 | 0 | 0.017 | 0.384 | 0.197 | 0.258 |
| 9 | 0 | 0 | 0 | 0 | 0 | 0 | 0 | 0 | 0 | 0.009 | 0.237 | 0.161 |
| 10 | 0 | 0 | 0 | 0 | 0 | 0 | 0 | 0 | 0 | 0 | 0.039 | 0.194 |
| 11 | 0 | 0 | 0 | 0 | 0 | 0 | 0 | 0 | 0 | 0 | 0 | 0 |

LAD

| Order n | Order m (parent segment) | | | | | | | | | | | |
|---|---|---|---|---|---|---|---|---|---|---|---|---|
| | 1 | 2 | 3 | 4 | 5 | 6 | 7 | 8 | 9 | 10 | 11 | 12 |
| 0 | 0 | 0.957 | 0.240 | 0.048 | 0 | 0 | 0 | 0 | 0 | 0 | 0 | 0 |
| 1 | 0 | 0.043 | 0.726 | 0.204 | 0.018 | 0 | 0 | 0 | 0 | 0 | 0 | 0 |
| 2 | 0 | 0 | 0.033 | 0.724 | 0.389 | 0.021 | 0.092 | 0.005 | 0 | 0 | 0 | 0 |
| 3 | 0 | 0 | 0 | 0.024 | 0.555 | 0.130 | 0.096 | 0.024 | 0.016 | 0.004 | 0 | 0 |
| 4 | 0 | 0 | 0 | 0 | 0.037 | 0.767 | 0.416 | 0.075 | 0.074 | 0.043 | 0.017 | 0 |
| 5 | 0 | 0 | 0 | 0 | 0 | 0.081 | 0.245 | 0.378 | 0.268 | 0.192 | 0.067 | 0 |
| 6 | 0 | 0 | 0 | 0 | 0 | 0 | 0.152 | 0.495 | 0.272 | 0.209 | 0.118 | 0 |
| 7 | 0 | 0 | 0 | 0 | 0 | 0 | 0 | 0.023 | 0.360 | 0.183 | 0.201 | 0.111 |
| 8 | 0 | 0 | 0 | 0 | 0 | 0 | 0 | 0 | 0.010 | 0.352 | 0.252 | 0.167 |
| 9 | 0 | 0 | 0 | 0 | 0 | 0 | 0 | 0 | 0 | 0.017 | 0.335 | 0.444 |
| 10 | 0 | 0 | 0 | 0 | 0 | 0 | 0 | 0 | 0 | 0 | 0.010 | 0.278 |
| 11 | 0 | 0 | 0 | 0 | 0 | 0 | 0 | 0 | 0 | 0 | 0 | 0 |

LCX

| Order n | Order m (parent segment) | | | | | | | | | | | |
|---|---|---|---|---|---|---|---|---|---|---|---|---|
| | 1 | 2 | 3 | 4 | 5 | 6 | 7 | 8 | 9 | 10 | 11 | 12 |
| 0 | 0 | 0.957 | 0.240 | 0.048 | 0 | 0 | 0 | 0 | 0 | 0 | 0 | 0 |
| 1 | 0 | 0.043 | 0.726 | 0.204 | 0.018 | 0 | 0 | 0 | 0 | 0 | 0 | 0 |
| 2 | 0 | 0 | 0.033 | 0.724 | 0.389 | 0.046 | 0 | 0 | 0 | 0 | 0 | 0 |
| 3 | 0 | 0 | 0 | 0.024 | 0.555 | 0.046 | 0.008 | 0.003 | 0 | 0 | 0 | 0 |
| 4 | 0 | 0 | 0 | 0 | 0.037 | 0.877 | 0.123 | 0.047 | 0.008 | 0 | 0 | 0 |
| 5 | 0 | 0 | 0 | 0 | 0 | 0.031 | 0.801 | 0.296 | 0.072 | 0.053 | 0 | 0 |
| 6 | 0 | 0 | 0 | 0 | 0 | 0 | 0.068 | 0.610 | 0.152 | 0.214 | 0.111 | 0 |
| 7 | 0 | 0 | 0 | 0 | 0 | 0 | 0 | 0.044 | 0.153 | 0.240 | 0.222 | 0 |
| 8 | 0 | 0 | 0 | 0 | 0 | 0 | 0 | 0 | 0.015 | 0.480 | 0.222 | 0 |
| 9 | 0 | 0 | 0 | 0 | 0 | 0 | 0 | 0 | 0.601 | 0.013 | 0.444 | 0 |
| 10 | 0 | 0 | 0 | 0 | 0 | 0 | 0 | 0 | 0 | 0 | 0 | 0 |
| 11 | 0 | 0 | 0 | 0 | 0 | 0 | 0 | 0 | 0 | 0 | 0 | 0 |

**Supplementary Section S4. Shortest distance of a point in the myocardium to endocardial surface.**

Let $\vec{p}=(x,y,z)\in R^3$ be a point in the myocarial tissue. Let the ellipsoid, whose origin is shifted to (0, 0, 0), be given by the polar parametrization

$$\vec{x}(\theta,\phi)=r\begin{pmatrix} a\cos\phi\cos\theta \\ b\cos\phi\sin\theta \\ c\sin\phi \end{pmatrix} \quad \text{Equation S4}$$

or equivalently by the equation

$$\left(\frac{x}{a}\right)^2+\left(\frac{y}{b}\right)^2+\left(\frac{z}{c}\right)^2=r^2 \quad \text{Equation S5}$$

A necessary condition for $\vec{x}$ to be the closest point to $\vec{p}$ is that $\vec{p}-\vec{x}$ is perpendicular to the tangent plane in $\vec{x}$, i.e.

$$(\vec{p}-\vec{x})\cdot\frac{\partial\vec{x}}{\partial\theta}=0 \quad and \quad (\vec{p}-\vec{x})\cdot\frac{\partial\vec{x}}{\partial\phi}=0 \quad \text{Equation S6}$$

which gives

$$\begin{aligned} 0&=(a^2-b^2)r\cos\theta\sin\theta\cos\phi-xa\sin\theta+yb\cos\theta \\ 0&=(a^2\cos^2\theta+b^2\sin^2\theta-c^2)r\sin\phi\cos\phi-xa\sin\phi\cos\theta-yb\sin\phi\sin\theta+zc\cos\phi \end{aligned} \quad \text{Equation S7}$$

The above system $0=F(\theta,\phi)$ can be solved with a Newton method, where one notes that $DF(\theta,\phi)=(a_{ij})\in R^{2x2}$ is given by

$$\begin{aligned} a_{11}&=r(a^2-b^2)(\cos^2\theta-\sin^2\theta)\cos\phi-xa\cos\theta-yb\sin\theta \\ a_{12}&=-r(a^2-b^2)\cos\theta\sin\theta\sin\phi \\ a_{21}&=-2r(a^2-b^2)\cos\theta\sin\theta\sin\phi\cos\phi+xa\sin\phi\sin\theta-yb\sin\phi\cos\theta \\ a_{22}&=r(a^2\cos^2\theta+b^2\sin^2\theta-c^2)(\cos^2\phi-\sin^2\phi)-xa\cos\phi\cos\theta-yb\cos\phi\sin\theta-zc\sin\phi \end{aligned}$$

Equation S8

with the initial guess

$$(\theta^{(0)},\phi^{(0)})=(\tan^{-1}(\frac{ay}{bx}),\tan^{-1}(z/[c((\frac{x}{a})^2+(\frac{y}{b})^2)^{\frac{1}{2}}]))\equiv(\arctan 2(ay,bx),\arctan 2(z,y((\frac{x}{a})^2+(\frac{y}{b})^2)^{\frac{1}{2}}))$$

(Equation S9)

Convergence of the Newton iterations of Equation S9 usually takes a small number of iterations. The desired distance is given by $|\vec{p}(x,y,z)-\vec{x}(\theta,\phi)|$.

**Section S5. Rotation matrix from axis and angle.**

Rotation of a point around a given axis is implemented using translation to origin, rotation for along global axis of choice, rotation in 2D, and reversing the operations. For some applications, it is helpful to be able to make a rotation with a given axis. Given a unit vector **u** = ($u_x$, $u_y$, $u_z$), where $u_x^2 + u_y^2 + u_z^2 = 1$, the matrix for a rotation by an angle of $\theta$ about an axis in the direction of **u** is

$$R=\begin{pmatrix} \cos\theta+u_x^2(1-\cos\theta) & u_xu_y(1-\cos\theta)-u_z\sin\theta & u_xu_z(1-\cos\theta)+u_y\sin\theta \\ u_xu_y(1-\cos\theta)+u_z\sin\theta & u_y^2(1-\cos\theta)+\sin\theta & u_yu_z(1-\cos\theta)-u_x\sin\theta \\ u_xu_z(1-\cos\theta)-u_y\sin\theta & u_yu_z(1-\cos\theta)+u_x\sin\theta & u_z^2(1-\cos\theta)+\sin\theta \end{pmatrix}$$

Equation S10.

This rotation matrix was used to estimate initial coordinates of daughter nodes at vasculature geometry construction stage.

## B. SUPPLEMENTARY RESULTS

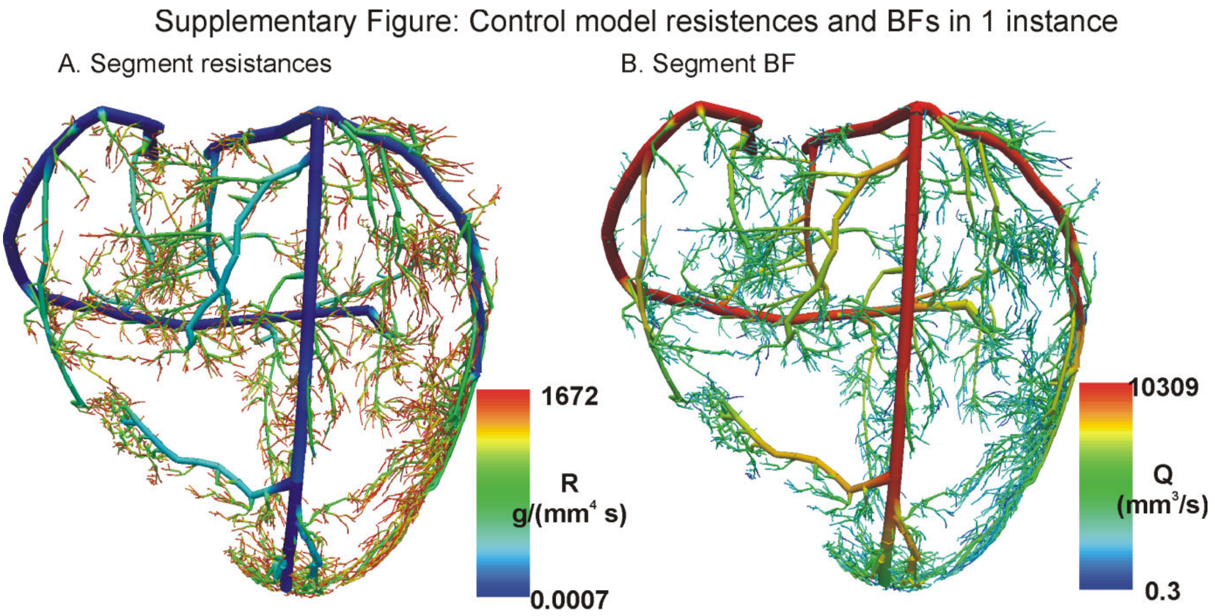


**Figure S2.** An instance of coronary vascular structure showing computed segment-by-segment resistances (left) and BF (right).

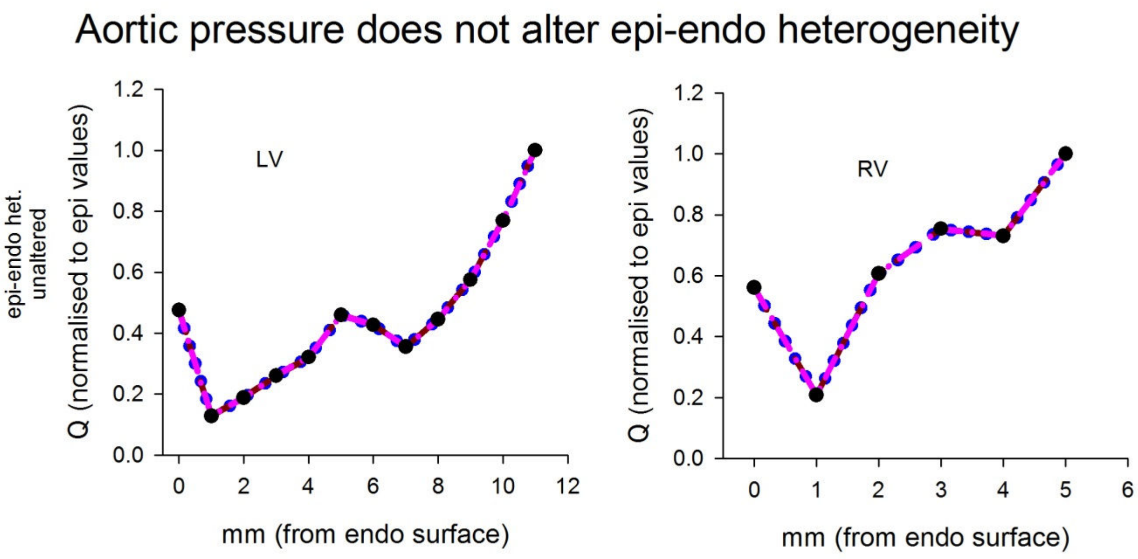


**Figure S3.** Transmural BF remains unchanged when aortic pressure is altered (see Figure 5 in main manuscript).

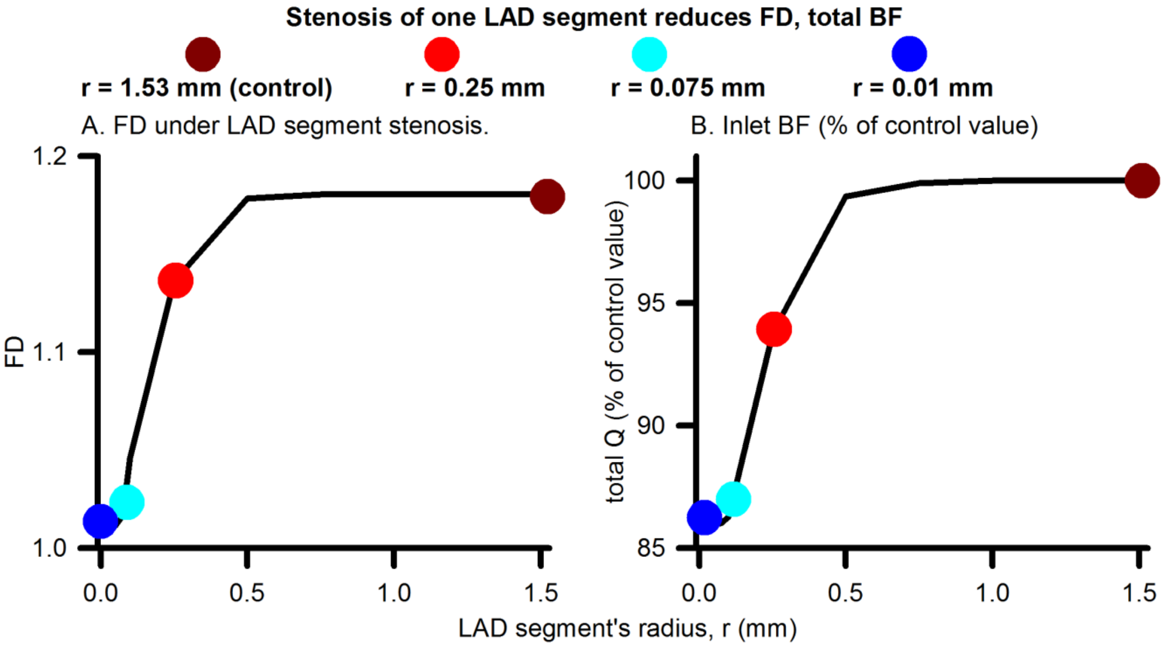


**Figure S4.** Effect of LAD segment stenosis on FD (A) and total BF (B).

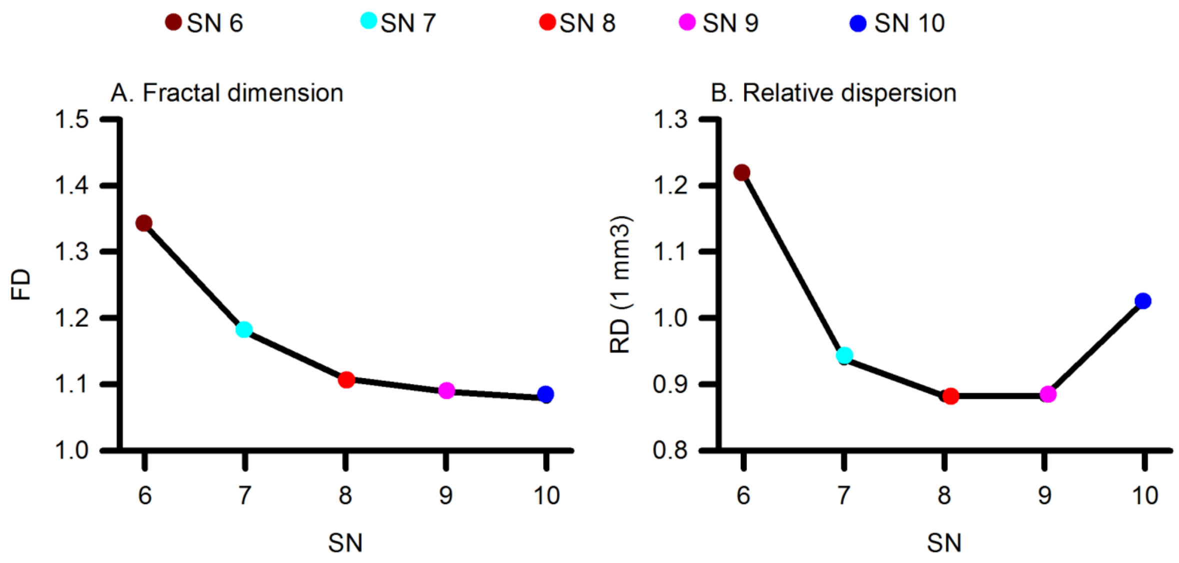


**Figure S5.** FD (A) and RD (B) when sub-trees of given SN were blocked.

## REFERENCES

Kassab, G.S., Rider, C.A., Tang, N.J., and Fung, Y.C. (1993). Morphometry of pig coronary arterial trees. Am J Physiol *265*, H350-365.
